# The Gut–Lung Axis in Allergic Asthma: A Narrative Review of Microbial Dysbiosis, Immune Regulation, and Nutritional Modulation

**DOI:** 10.3390/nu18091336

**Published:** 2026-04-23

**Authors:** Chi-Kun Chiang, Ching-Long Lai, Ming-Huang Chiu, Chi-Jung Huang

**Affiliations:** 1 Division of Gastroenterology, Department of Internal Medicine, Cathay General Hospital, Taipei City 106438, Taiwan; hsetnja@gmail.com; 2 Division of Basic Medical Sciences, Department of Nursing, Chang Gung University of Science and Technology, Taoyuan City 333324, Taiwan; dinolai@mail.cgust.edu.tw; 3 Center for Drug Research and Development, Chang Gung University of Science and Technology, Taoyuan City 333324, Taiwan; 4 Division of Pulmonology & Respiratory Care, Department of Internal Medicine, Cathay General Hospital, Taipei City 106438, Taiwan; 5 Department of Medical Research, Cathay General Hospital, Taipei City 106438, Taiwan; 6 Department of Biochemistry, National Defense Medical Center, Taipei City 114201, Taiwan

**Keywords:** allergic asthma, gut–lung axis, gut microbiota, microbial dysbiosis, short-chain fatty acids, immune tolerance, early-life microbial exposure, precision nutrition

## Abstract

Allergic asthma is a prevalent chronic inflammatory disease of the airways whose pathogenesis has traditionally been attributed to localized immune dysfunction within the lung. However, accumulating evidence from microbiome research supports a broader system-level perspective in which cross-organ interactions contribute to disease susceptibility and progression. In particular, the gut–lung axis has emerged as a key regulatory pathway linking intestinal microbial ecology, immune development, and respiratory health. This review synthesizes current epidemiological, mechanistic, and experimental evidence supporting the role of gut microbiota dysbiosis in allergic asthma. We examine how early-life environmental and nutritional exposures and gut microbiota establishment during critical developmental windows shape long-term immune tolerance and asthma susceptibility. We then summarize characteristic features of asthma-associated gut dysbiosis and discuss how microbial-derived metabolites, including short-chain fatty acids, tryptophan metabolites, pro-allergic lipid mediators such as 12,13-dihydroxy-9Z-octadecenoic acid, and bacterial-derived histamine, modulate distal airway immune responses through epigenetic, receptor-mediated, and immune trafficking mechanisms. Particular emphasis is placed on the role of diet as a key upstream regulator of gut microbiota composition and metabolic function. Finally, we evaluate experimental and translational studies targeting the gut–lung axis, including dietary modulation, microbiome-targeted interventions such as fecal microbiota transplantation, and emerging postbiotic approaches. Collectively, current evidence indicates that gut microbial composition and metabolic function are critical determinants of respiratory immune homeostasis. Targeting the gut–lung axis through nutrition- and microbiome-based strategies offers a promising avenue for the prevention and precision treatment of allergic asthma.

## 1. Introduction

Allergic asthma is a heterogeneous chronic respiratory disease characterized by recurrent wheezing, dyspnea, chest tightness, and coughing [1,2]. Its pathological hallmarks include airway hyperresponsiveness, mucus hypersecretion, airway remodeling, and a predominantly T helper 2 (Th2)-driven inflammatory response [3]. Asthma affects hundreds of millions of individuals globally, and its prevalence has increased substantially over recent decades, particularly in industrialized countries, representing a major and growing public health challenge [4,5,6].

Historically, asthma has been viewed as a localized pulmonary disorder resulting from allergen sensitization and the accumulation of effector immune cells, such as eosinophils, mast cells, and Th2 lymphocytes, within the airways [7]. However, therapies primarily targeting airway inflammation, including inhaled corticosteroids, are insufficient for a considerable proportion of patients and fail to account for the striking rise in asthma incidence associated with modern environmental and lifestyle changes [8]. These limitations have driven a shift toward identifying systemic factors beyond the lung that contribute to disease susceptibility and persistence. Given the central role of diet in shaping gut microbiota composition and function, nutritional factors represent a critical and modifiable component of the gut–lung axis.

## 2. Methodology and Analysis

### 2.1. Literature Search Strategy

A structured literature search was conducted to identify studies examining the role of the gut–lung axis in allergic asthma, with particular emphasis on gut microbiota dysbiosis, immune regulation, microbial-derived metabolites, and microbiome- or nutrition-based interventions. Electronic searches were performed in PubMed/MEDLINE, Web of Science, and Scopus for articles published up to December 2025. Search terms were constructed using combinations of keywords related to asthma and gut microbiota, including “allergic asthma”, “gut–lung axis”, “gut microbiota”, “microbial dysbiosis”, “short-chain fatty acids”, “tryptophan metabolism”, “immune tolerance”, “early-life microbial exposure”, “dietary fiber”, “probiotics, postbiotics”, and “fecal microbiota transplantation”. Boolean operators were applied to refine the search strategy. In addition, the reference lists of relevant reviews and primary research articles were manually screened to identify further eligible studies. Only English-language publications were included, and both human and experimental animal studies were considered to capture epidemiological, mechanistic, and translational evidence.

### 2.2. Literature Overview Approach

This review was conducted as a narrative overview and did not follow a formal systematic review or meta-analysis protocol. Studies were selected based on their relevance to the gut–lung axis, methodological quality, and contribution to advancing a mechanistic or translational understanding of allergic asthma. Particular attention was given to longitudinal birth cohort studies, epidemiological investigations, mechanistic experimental work, and translational research addressing early-life microbial exposure, immune programming, and microbiota-targeted interventions. Rather than attempting exhaustive coverage, this review emphasizes conceptual coherence, reproducibility of findings across independent studies, and biological plausibility. It is important to note that the characterization of gut microbiota in asthma varies depending on the sequencing approach used. Studies based on 16S rRNA gene sequencing, which are influenced by the choice of variable region and generally provide genus-level resolution, may yield different taxonomic profiles compared to shotgun metagenomic approaches, which offer higher taxonomic and functional resolution. This methodological heterogeneity may partly explain inconsistencies across studies and should be considered when interpreting reports of “consistent” microbial signatures in allergic asthma.

### 2.3. Analytical Approach

An integrative analytical framework was applied to synthesize evidence across microbial, immunological, metabolic, and clinical domains. Findings were analyzed thematically to identify convergent mechanisms linking gut microbiota composition and function with immune regulation and allergic airway disease. Emphasis was placed on the temporal specificity of early-life microbial influences, functional alterations in microbial-derived metabolites, immune signaling pathways mediating gut–lung communication, and the translational implications of microbiome- and nutrition-based interventions. This approach aims to provide a mechanistically grounded synthesis that situates allergic asthma within a systemic, microbiota-informed disease framework.

## 3. Early-Life Microbial Exposure and Immune Programming

Early-life exposure to environmental microbes plays a decisive role in shaping immune tolerance and asthma susceptibility, primarily through its influence on gut microbiota establishment during critical developmental windows. The interpretation of associations between early-life exposure and asthma risk requires caution, as many of these relationships are subject to substantial confounding and bias. Factors such as socioeconomic status, parental atopy, healthcare utilization, urbanicity, diet, and infection burden may influence both exposure and outcome. In addition, certain exposures, particularly antibiotic use in infancy, are highly susceptible to confounding by indication, whereby underlying infections rather than the exposure itself may drive the observed associations. Reverse causation and differential healthcare-seeking behaviors may further complicate interpretation.

### 3.1. Environmental Microbial Exposure and the Hygiene Paradigm

Epidemiological studies suggest that early-life environmental microbial exposure is associated with asthma risk, though results are heterogeneous [9]. The historically termed “Hygiene Hypothesis” has largely been superseded by the “Old Friends” and “Biodiversity” hypotheses, which more accurately emphasize the role of exposure to diverse, co-evolved microbial communities in immune development [10,11,12].

Among the most frequently cited observations is the “farm effect”, in which children raised in traditional farming environments exhibit lower rates of asthma and allergic disease. These protective associations have been linked to increased environmental microbial diversity; non-pathogenic microbial exposure in high-diversity, non-pathogenic settings, as well as exposure to pathogenic or dysbiotic microbial communities, such as those encountered in conditions of poor sanitation or following environmental disruptions like flooding, has been associated with gut microbiota imbalance and adverse health outcomes [13].

Importantly, the interpretation of these associations requires caution as they are subject to substantial confounding by socioeconomic status, lifestyle factors, parental atopy, infection burden, and healthcare access. Together, these findings suggest that it is not microbial exposure per se, but the composition, diversity, and ecological context of microbial communities that shape immune development and asthma susceptibility.

Large multinational cohort studies, including the International Study of Asthma and Allergies in Childhood (ISAAC) and the Multidisciplinary Study to Identify the Genetic and Environmental Causes of Asthma in the European Community Advanced Study (also named GABRIEL), suggest that children raised in traditional farming environments exhibit significantly lower prevalence of asthma and other allergic diseases compared with non-farm counterparts living in the same regions [14,15,16,17]. A landmark comparative study of Amish and Hutterite children further clarified the underlying mechanisms [18]. Despite similar genetic backgrounds, Amish children, who experience continuous exposure to livestock-associated microbes, display markedly lower asthma prevalence than Hutterite children, whose industrialized farming practices limit such exposure. Environmental analyses revealed higher microbial diversity and endotoxin levels in Amish households, and the experimental administration of Amish farm dust conferred protection against allergic airway inflammation in murine models via Toll-like receptor 4-dependent innate immune pathways [18].

In urban settings, domestic animals represent an important source of microbial enrichment. Although pet dander is a known allergen, associations between pet exposure and asthma risk are complex and context-dependent, with evidence suggesting both protective and risk-modifying effects depending on timing, microbial environment, and host susceptibility [19]. The Urban Environment and Childhood Asthma (URECA) cohort further demonstrated that allergen exposure in the context of high microbial diversity promotes immune tolerance, whereas allergen exposure in microbially impoverished environments increases sensitization risk [20]. Collectively, these findings suggest that environmental microbial richness, rather than allergen avoidance alone, is critical for shaping non-allergic immune trajectories.

### 3.2. Establishment of Gut Microbiota During the Critical Developmental Window

Environmental microbial exposures exert their long-term effects on asthma susceptibility largely through the modulation of gut microbiota establishment during early life. A growing body of evidence supports the existence of a “critical developmental window,” spanning approximately the first months to year of life, during which gut microbial composition and metabolic activity exert a disproportionate influence on immune system maturation [21,22]. Perturbations during this period may result in persistent defects in immune tolerance that are not fully reversible later in life.

Strong support for this concept comes from the Canadian Healthy Infant Longitudinal Development cohort, which demonstrated that infants who later developed asthma exhibited significant depletion of specific gut bacterial taxa, *Faecalibacterium*, *Lachnospira*, *Veillonella*, and *Rothia* (collectively termed the FLVR group), as early as three months of age [23]. This dysbiotic signature was accompanied by reduced fecal acetate concentrations, indicating impaired microbial metabolic function. Notably, although microbial composition partially normalized by one year of age, early-life dysbiosis alone was sufficient to predict asthma risk at school age, underscoring the temporal specificity of immune education [23].

Several perinatal and postnatal factors critically shape gut microbiota assembly during this window. Mode of delivery is a significant factor, with vaginal birth enabling the transmission of maternal vaginal and intestinal microbes such as *Lactobacillus* and *Prevotella*. In contrast, cesarean section is linked to delayed microbial colonization and has been correlated with a slight elevation in asthma risk. Nevertheless, findings from sibling comparison and registry-based studies have notably reduced these associations, indicating that shared familial and environmental confounding may play an important role [24,25]. Feeding practices further modulate microbial development. Breast milk supplies not only nutrients, but also a diverse array of bioactive components, including human milk oligosaccharides, secretory IgA, maternal microbes, lactoferrin, and lysozyme, which collectively promote beneficial microbial colonization, enhance mucosal barrier function, and support immune tolerance [26]. In contrast, early-life antibiotic exposure represents one of the most potent disruptors of gut microbial ecology, leading to reduced diversity and altered metabolite production. Experimental studies demonstrate that neonatal, but not adult, antibiotic exposure markedly exacerbates allergic airway inflammation later in life. However, although early-life antibiotic exposure has been associated with increased asthma risk in human studies, these findings are difficult to interpret due to confounding by indication and underlying infection-related risk [27].

**Figure 1:**
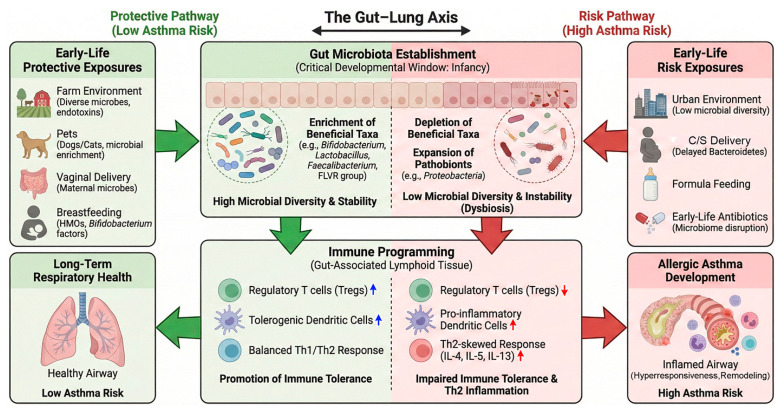
Early-life programming of the gut–lung axis in allergic asthma. This schematic illustrates how environmental and perinatal factors during the “critical developmental window” of infancy shape gut microbiota composition and long-term respiratory health. ((**Left**), Protective Pathway) Exposures such as farm environments, pets, vaginal delivery, and breastfeeding promote high microbial diversity and the colonization of beneficial taxa (e.g., *Bifidobacterium*, *Faecalibacterium*). This factors support immune tolerance through the induction of regulatory T cells and tolerogenic dendritic cells, thereby protecting against airway inflammation and reducing asthma risk. (**(Middle)**, Gut microbiota establishment and immune programming) During the critical developmental window of infancy, gut microbiota composition is shaped by environmental and perinatal factors. A balanced microbiota, including enrichment of short-chain fatty acid-producing bacteria (e.g., the FLVR group), promotes immune homeostasis and tolerance. In contrast, reduced diversity and depletion of beneficial microbes impair immune maturation, leading to dysregulated immune responses. ((**Right**), Risk Pathway) Conversely, factors such as cesarean section, early-life antibiotic use, and lack of microbial exposure lead to dysbiosis, characterized by reduced diversity and the expansion of pathobionts (e.g., Proteobacteria). This dysbiotic state disrupts immune maturation, skewing the immune system toward a T helper 2 (Th2)-driven inflammatory response and increasing susceptibility to allergic asthma. HMOs: human milk oligosaccharides; FLVR group: *Faecalibacterium*, *Lachnospira*, *Veillonella*, and *Rothia* genera; C/S: cesarean section; Blue arrow; positive change; Red arrow: negative change.

Together, these findings establish early-life environmental exposure and gut microbiota assembly as tightly linked processes that program immune tolerance and asthma susceptibility through temporally constrained developmental mechanisms. Key early-life environmental and perinatal factors influencing gut microbiota establishment and asthma risk are summarized in Table 1, while a schematic overview of early-life environmental exposures, gut microbiota establishment, and immune programming along the gut–lung axis is shown in Figure 1.

## 4. Gut Microbiota Dysbiosis in Allergic Asthma

Accumulating evidence indicates that allergic asthma is associated with gut microbiota dysbiosis, characterized by both structural and functional alterations in the intestinal microbial ecosystem [28]. Rather than representing a nonspecific loss of microbial balance, asthma-associated dysbiosis involves reproducible changes in microbial diversity, the depletion of protective commensal taxa, and the expansion of microorganisms with pro-allergic or pro-inflammatory potential.

### 4.1. Reduced Microbial Diversity and Ecosystem Instability

One of the most reported features of gut microbiota dysbiosis in allergic asthma is a significant reduction in α-diversity, reflecting decreased species richness within individual hosts [29]. High microbial diversity is generally associated with ecological stability, metabolic redundancy, and resilience to perturbations. In contrast, reduced diversity signifies a fragile ecosystem that is less capable of maintaining immune homeostasis [21]. Longitudinal cohort studies demonstrate that diminished gut microbial diversity in early infancy precedes the development of asthma later in childhood, suggesting that diversity loss is not merely a consequence of disease, but an early marker of asthma susceptibility [29].

### 4.2. Depletion of Protective Commensal Taxa

Beyond overall diversity, allergic asthma is associated with the selective depletion of specific bacterial taxa with known immunoregulatory functions. Among these, *Faecalibacterium prausnitzii*, a dominant member of the healthy adult gut microbiota and a major producer of short-chain fatty acids (SCFAs), is reduced in both pediatric and adult asthma patients [30]. Experimental studies further support a protective role for *F. prausnitzii*, as its absence correlates with increased airway inflammatory markers and heightened disease severity [30,31].

*Akkermansia muciniphila*, a mucin-degrading bacterium critical for maintaining intestinal barrier integrity, is also reduced in children with allergic asthma [30]. Decreased abundance of *A. muciniphila* has been linked to compromised gut barrier function, which may facilitate systemic exposure to microbial products and dietary antigens. Similarly, *Bifidobacterium* species, particularly important during early life for immune tolerance induction, are underrepresented in individuals with persistent asthma [23,32,33,34]. The collective loss of these commensal taxa reflects a shift away from a microbiota configuration that supports immune regulation.

### 4.3. Expansion of Pathobionts and Pro-Allergic Functional Signatures

In parallel with the loss of beneficial microbes, asthma-associated dysbiosis is marked by the expansion of pathobionts and functional microbial traits that favor allergic inflammation. Notably, histamine-secreting bacteria such as *Morganella morganii* are enriched in the gut microbiota of asthma patients [35]. These organisms possess histidine decarboxylase activity and contribute to elevated intestinal and systemic histamine levels, which have been linked to increased asthma severity.

An increased relative abundance of Proteobacteria, including *Escherichia coli*, has also been reported in asthma cohorts [36,37]. Proteobacteria expansion is widely regarded as a hallmark of dysbiosis and is associated with increased inflammatory potential, particularly in the context of impaired gut barrier function. In addition, metagenomic analyses have identified the enrichment of bacterial genes encoding epoxide hydrolases responsible for producing the lipid metabolite 12,13-dihydroxy-9Z-octadecenoic acid (12,13-diHOME) in neonates at high risk for asthma [38]. This functional shift highlights that dysbiosis in asthma involves not only taxonomic alterations, but also changes in microbial metabolic capacity.

Taken together, these findings indicate that gut microbiota dysbiosis in allergic asthma is defined by a coordinated loss of immune-supportive commensals and the enrichment of microbial functions that favor pro-allergic immune environments, thereby creating conditions permissive for the development and persistence of airway inflammation. The principal taxonomic and functional features of gut microbiota dysbiosis associated with allergic asthma are summarized in Table 2.

## 5. Mechanistic Basis of the Gut–Lung Axis

The gut–lung axis operates through a complex network of immune and metabolic signaling pathways by which intestinal microbial states are translated into distal airway immune responses [40,41,42]. Central to this inter-organ communication are microbial-derived metabolites and immune cell trafficking mechanisms that collectively regulate immune tolerance and inflammatory balance in the lung.

### 5.1. SCFAs and Epigenetic Regulation of Type 2 Immunity

SCFAs, primarily acetate, propionate, and butyrate, are among the most extensively characterized microbial metabolites mediating gut–lung immune crosstalk [40]. Produced through the bacterial fermentation of dietary fiber, SCFAs exert broad anti-inflammatory effects by modulating both innate and adaptive immune responses relevant to allergic asthma.

SCFAs signal through G protein-coupled receptors, including GPR43 and GPR41, expressed on immune cells such as eosinophils, dendritic cells, and regulatory T (Treg) cells. The activation of these receptors suppresses eosinophil chemotaxis and degranulation, promotes tolerogenic dendritic cell phenotypes, and enhances Treg differentiation, collectively dampening type 2 inflammatory responses in the airway [40]. In parallel, SCFAs, particularly butyrate, function as histone deacetylase (HDAC) inhibitors, thereby exerting epigenetic control over immune gene expression. In innate lymphoid cells type 2, the butyrate-mediated inhibition of HDAC3 reduces the transcription of interleukin (IL)-5 and IL-13 while enhancing acetylation at regulatory loci associated with immune tolerance [43,44,45].

Experimental evidence further supports the central role of SCFAs in asthma pathophysiology. The depletion of intestinal SCFAs exacerbates airway inflammation and hyperresponsiveness, whereas the restoration of SCFA levels, either through dietary intervention or FMT, attenuates lung inflammation and airway remodeling [46]. These findings position SCFAs as key molecular mediators linking diet, gut microbiota composition, and allergic airway disease.

Dietary factors play a central role in shaping gut microbiota composition and metabolic output, thereby influencing the gut–lung axis. In particular, dietary fiber intake promotes the production of SCFAs by commensal bacteria, linking nutritional exposure directly to immunoregulatory pathways involved in allergic asthma [40,41]. Conversely, Western-style diets low in fiber and high in fat may contribute to dysbiosis and the reduced production of beneficial metabolites, thereby impairing immune tolerance [42].

### 5.2. Tryptophan Metabolism and Aryl Hydrocarbon Receptor Signaling

Beyond SCFAs, gut microbial metabolism of dietary tryptophan represents another critical regulatory axis influencing lung immunity. Specific bacterial taxa, including *Lactobacillus* species, convert tryptophan into indole derivatives such as indole-3-acetic acid, indole, and indole-3-propionic acid [47]. These metabolites act as ligands for the aryl hydrocarbon receptor (AhR), a transcription factor expressed on multiple immune cell types.

The activation of AhR signaling promotes the differentiation and function of type 3 innate lymphoid cells and enhances the production of IL-22, a cytokine essential for maintaining mucosal barrier integrity and regulating tissue repair responses [47]. Through this pathway, gut-derived tryptophan metabolites indirectly support epithelial homeostasis and immune balance in the lung, reducing susceptibility to allergen-induced inflammation. The disruption of microbial tryptophan metabolism therefore represents an additional mechanism by which gut dysbiosis may impair systemic immune tolerance.

### 5.3. Immune Cell Trafficking Between Gut and Lung

In addition to soluble metabolites, the gut–lung axis is reinforced by the physical trafficking of immune cells between mucosal sites. Immune cells primed in the gut-associated lymphoid tissue, particularly Treg cells, can migrate to the lung via shared chemokine and homing receptor pathways [41,42]. Following activation in the intestinal environment, these cells acquire tissue-specific homing properties after gut priming; however, the precise mechanisms governing their migration to the lung remain incompletely defined.

Once in the lung, gut-educated immune cells contribute to the suppression of excessive allergic inflammation by inhibiting effector T cell responses and maintaining immune tolerance at the airway mucosa. This cellular migration provides a direct immunological conduit through which intestinal immune programming exerts long-range effects on respiratory immunity.

### 5.4. Pro-Allergic Microbial Metabolites: 12,13-diHOME and Bacterial-Derived Histamine

While many microbial metabolites promote immune tolerance, gut dysbiosis can also favor the production of metabolites that actively disrupt regulatory pathways and promote allergic inflammation. One such example is 12,13-diHOME, a linoleic acid-derived lipid metabolite produced by bacterial epoxide hydrolases. Elevated fecal levels of 12,13-diHOME have been detected in neonates at high risk for asthma and are associated with impaired immune tolerance [38].

Mechanistically, 12,13-diHOME reduces both the abundance and suppressive function of Treg cells and alters metabolic programming in dendritic cells, thereby creating an immune milieu permissive for allergic sensitization. In parallel, the enrichment of histamine-producing gut bacteria, such as *Morganella morganii*, leads to increased intestinal histamine production [35]. This bacterial-derived histamine can enter systemic circulation and act synergistically with host-derived histamine to exacerbate airway inflammation through immune modulation via histamine H4 receptors and promote bronchoconstriction primarily through H1 receptor-mediated effects on airway smooth muscle.

**Figure 2:**
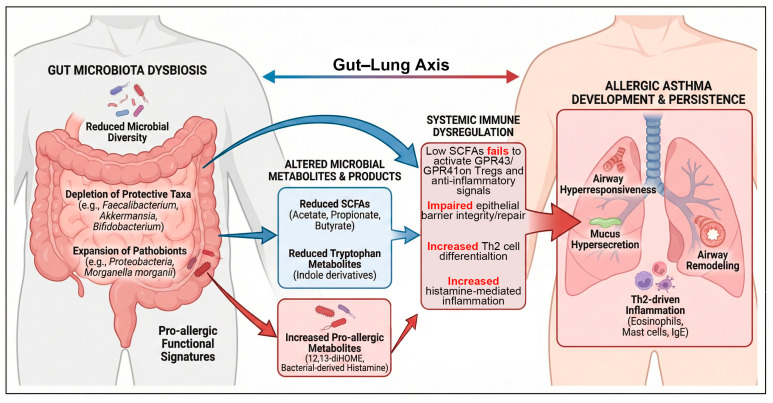
Mechanistic pathways linking gut microbiota dysbiosis to allergic asthma. This diagram illustrates the bidirectional immune and metabolic crosstalk mediating the gut–lung axis. (**Left**) Gut microbiota dysbiosis involves a loss of microbial diversity, the depletion of protective taxa (e.g., *Faecalibacterium*, *Bifidobacterium*), and the expansion of pathobionts (e.g., *Proteobacteria*). (**Middle**) This imbalance alters the profile of microbial-derived metabolites and immune signals. Protective pathways (blue arrows) include the production of short-chain fatty acids (SCFAs; acetate, propionate, butyrate) and tryptophan metabolites (indole derivatives). These metabolites promote immune tolerance by inhibiting histone deacetylases (HDACs), activating G protein-coupled receptors (GPRs) on regulatory T cells, and stimulating aryl hydrocarbon receptor (AhR) signaling. Risk pathways (red arrows) involve the secretion of pro-allergic metabolites such as 12,13-diHOME and bacterial-derived histamine, which disrupt Treg function and promote systemic inflammation. (**Right**) Gut-educated immune cells migrate to the respiratory tract via circulation. The net result of these interactions determines the lung immune tone; dysbiosis-driven signaling favors a Th2-skewed inflammatory response, leading to airway hyperresponsiveness, mucus hypersecretion, and airway remodeling characteristic of allergic asthma.

Together, these findings underscore the fact that gut microbial metabolites exert bidirectional control over lung immunity, with the balance between tolerogenic and pro-allergic signals determining susceptibility to allergic asthma. Major microbial-derived metabolites mediating gut–lung immune communication and their immunological effects are summarized in Table 3, while the key metabolic and immunological pathways underlying gut–lung communication in allergic asthma are illustrated in Figure 2.

## 6. Respiratory Microbiota as a Downstream Target of Gut Dysbiosis

The long-standing concept of the lung as a sterile organ has been overturned by culture-independent sequencing approaches, which reveal that the respiratory tract harbors a low-biomass but biologically meaningful microbial community [48]. In the context of allergic asthma, alterations in the respiratory microbiota are increasingly recognized as downstream consequences of systemic immune dysregulation shaped by early-life microbial exposure and gut microbiota composition.

Early-life airway colonization patterns are particularly predictive of asthma development. Prospective birth cohort studies have demonstrated that nasopharyngeal colonization by pathogenic bacteria such as *Streptococcus pneumoniae*, *Moraxella catarrhalis*, and *Haemophilus influenzae* during infancy is strongly associated with recurrent wheeze and increased asthma risk later in childhood [49]. These findings suggest that early microbial–immune interactions at the airway mucosa can amplify pre-existing immune susceptibility established through gut microbiota-mediated programming.

In established asthma, both pediatric and adult patients exhibit reproducible features of respiratory dysbiosis. A relative expansion of Proteobacteria in bronchial lavage and sputum samples correlates with increased airway hyperresponsiveness, neutrophilic inflammation, and disease severity [36,37,50]. Notably, colonization with *Haemophilus* species is associated with corticosteroid resistance and poorer clinical outcomes, highlighting a clinically relevant interaction between airway microbial composition and treatment response [37,51,52,53].

Rather than functioning independently, the respiratory microbiota likely reflects the integrated effects of microbial immigration from the upper airway, local environmental conditions, and systemic immune tone shaped by the gut–lung axis [54]. Gut-derived immune signals and metabolites influence airway epithelial integrity and immune responsiveness, thereby determining which microbial communities can persist in the lower airways. In this framework, respiratory dysbiosis is best viewed as a secondary manifestation of upstream immune and metabolic perturbations rather than a primary driver of disease.

Together, current evidence supports a model in which gut microbiota dysbiosis and impaired immune tolerance create a permissive environment for pathological airway microbial colonization, which, in turn, exacerbates inflammation and contributes to asthma severity and treatment resistance.

## 7. Experimental and Clinical Evidence

The mechanistic links between gut microbiota dysbiosis and allergic asthma raise an essential translational question: can the restoration of gut microbial composition and metabolic function reverse established airway pathology? Recent experimental and interventional studies provide increasing support for this concept, positioning the gut–lung axis as a promising therapeutic target.

### 7.1. Experimental Evidence (Animal/FMT)

Among microbiome-based interventions, FMT offers the most direct experimental approach to testing causality within the gut–lung axis. Lai et al. provided compelling evidence using an ovalbumin-induced asthmatic rat model, demonstrating that the transplantation of fecal microbiota from healthy donors significantly ameliorated multiple hallmarks of allergic asthma [46].

Compared with untreated asthmatic controls and recipients of microbiota from asthmatic donors, rats receiving healthy donor FMT exhibited marked improvements in lung function parameters, including peak expiratory flow and airway resistance. These functional benefits were accompanied by substantial reductions in eosinophilic infiltration in bronchoalveolar lavage fluid and the downregulation of key type 2 cytokines (IL-4, IL-5, and IL-13), as well as epithelial alarmins such as IL-33 and thymic stromal lymphopoietin. Importantly, histological analyses revealed the significant attenuation of collagen deposition and airway remodeling, indicating that gut microbiota restoration may influence not only inflammation, but also structural disease features.

At the metabolic level, FMT restored intestinal SCFA concentrations that were profoundly depleted in asthmatic animals, and the normalization of these metabolites closely correlated with improved pulmonary outcomes [46]. Together, these findings provide direct experimental evidence that gut microbial composition and metabolic output play a causal role in regulating airway inflammation and remodeling, establishing FMT as a powerful proof-of-concept for the gut–lung axis.

### 7.2. Translational and Clinical Studies (Diet, Probiotics, Humans)

While FMT is valuable experimentally, its clinical application in allergic asthma is limited by safety concerns, donor variability, and lack of standardization [55]. Consequently, attention has shifted toward more defined and controllable microbiome-targeted strategies.

Dietary modulation represents the most accessible intervention. High-fiber diets increase the endogenous production of SCFAs by commensal bacteria and have been shown in animal models to suppress allergic airway inflammation and enhance immune tolerance via the gut–lung axis [40]. These findings highlight dietary fiber as a modifiable environmental factor with therapeutic potential. Beyond fiber, broader dietary patterns and bioactive food compounds may further shape gut microbial composition and metabolic output. However, current evidence in humans remains limited and heterogeneous, underscoring the need for mechanistic studies and well-controlled clinical trials [56].

In addition to general dietary patterns, fermented foods represent a distinct nutritional component that provides live microorganisms and bioactive metabolites [57]. Foods such as yogurt, kefir, kimchi, and fermented soy products contain complex microbial consortia that differ fundamentally from conventional probiotic supplements, which typically involve selected strains with defined composition [57]. These foods may influence host immunity through multiple mechanisms, including transient microbial colonization, metabolite production (e.g., SCFAs), and the modulation of intestinal barrier function [57,58]. Despite these plausible mechanisms, direct evidence linking fermented food consumption to allergic asthma remains limited, with most data derived from observational studies or extrapolated from other immune-mediated conditions, highlighting the need for targeted clinical investigation [56].

Probiotic supplementation has shown no consistent evidence of clinical benefit in human asthma trials, likely reflecting strain-specific effects and the limited colonization capacity of conventional probiotics such as *Lactobacillus* and *Bifidobacterium* species. Clinical trials evaluating probiotics in asthma have yielded inconsistent results, with no reproducible evidence of meaningful clinical benefit [59,60]. Notably, even in well-studied gut–brain axis conditions such as irritable bowel syndrome, probiotics have demonstrated limited or inconsistent clinical efficacy [61], highlighting the need for more targeted and mechanism-based approaches. In contrast, next-generation probiotics, defined by functional properties rather than taxonomic identity, are emerging as promising candidates. Notably, *F. prausnitzii* has attracted interest due to its potent anti-inflammatory activity and capacity to restore key microbial metabolites [30,31].

Beyond live microbes, postbiotic approaches targeting specific microbial metabolites or signaling pathways are gaining traction. These include the direct supplementation of SCFAs or modulation of pro-allergic metabolites such as 12,13-diHOME. In parallel, the development of rationally designed microbial consortia, such as combinations of early-life protective taxa identified in longitudinal cohorts, may offer a safer and more reproducible alternative to conventional FMT [23,46]. Current experimental and translational strategies targeting the gut–lung axis are summarized in Table 4.

### 7.3. Toward Precision Nutrition and Microbiome-Guided Therapy

Advances in microbiome profiling and metabolomics are paving the way for precision approaches to asthma prevention and treatment. Rather than applying uniform interventions, future strategies are likely to tailor dietary, probiotic, or postbiotic therapies based on individual microbial and metabolic signatures, such as reduced butyrate production or the enrichment of pro-allergic lipid metabolites [56]. Within this framework, the gut microbiome serves both as a therapeutic target and a biomarker for disease stratification and treatment response.

Collectively, experimental and translational evidence supports the feasibility of modulating the gut–lung axis to attenuate allergic airway disease. Although clinical translation remains in its early stages, microbiome-informed nutritional and microbial interventions represent a promising frontier for the precision management of allergic asthma.

## 8. Conclusions and Future Directions

These findings highlight the fact that diet represents a key upstream modulator of the gut–lung axis, linking environmental exposure, microbial metabolism, and immune regulation in allergic asthma. Collectively, current evidence supports a conceptual shift in allergic asthma from a strictly lung-centered disorder to a systemic disease shaped by gut microbiota-driven immune regulation. Across epidemiological studies, longitudinal birth cohorts, mechanistic investigations, and experimental interventions, a suggested pattern emerges in which early-life microbial exposure and gut microbiota establishment critically determine long-term respiratory immune outcomes.

Asthma-associated dysbiosis is not limited to reduced microbial diversity but involves the coordinated loss of immunoregulatory commensal taxa and enrichment of microbial functions that favor allergic inflammation. Microbial-derived metabolites play a central role in mediating gut–lung communication, with SCFAs promoting immune tolerance through receptor signaling and epigenetic regulation, while pro-allergic metabolites such as 12,13-diHOME and bacterial-derived histamine disrupt regulatory pathways and exacerbate airway inflammation. From a clinical gastroenterological perspective, the systemic impact of these metabolites is fundamentally governed by intestinal barrier integrity. The balance between these opposing microbial signals appears to be a key determinant of asthma susceptibility, severity, and treatment responsiveness.

Experimental studies, particularly FMT in animal models, provide direct causal evidence that restoring gut microbial structure and metabolic output can reverse airway inflammation and remodeling. These findings support the feasibility of targeting the gut–lung axis through nutrition-based and microbiome-informed interventions. Future therapeutic strategies, ranging from high-fiber diets and next-generation probiotics to specific prebiotic/postbiotic combinations, and rationally designed microbial consortia should focus on reinforcing the mucosal barrier alongside taxonomic restoration. This dual approach elevates gut–lung axis interventions from simple microbial supplementation to a systemic enhancement of mucosal immune defense.

Importantly, these mechanisms are primarily derived from studies of allergic (type 2–high) asthma and early-life sensitization. In contrast, T2-low and neutrophilic asthma represent biologically distinct endotypes characterized by Th1/Th17-skewed inflammation, distinct microbial patterns, and differential treatment responses, and may not be adequately explained by the gut–lung axis mechanisms described here.

Consistent with this distinction, accumulating evidence indicates that the gut–lung axis shapes distinct immune phenotypes in asthma [63,64]. Microbial metabolites, particularly SCFAs, regulate T2-high eosinophilic inflammation by modulating Th2/Th17 balance, whereas dysbiosis is increasingly associated with T2-low neutrophilic asthma characterized by Th1/Th17 polarization and reduced corticosteroid responsiveness. Decreased SCFA production may impair Treg-cell function and promote IL-6/IL-17-driven inflammatory pathways, thereby favoring non-type 2 airway inflammation. Although direct clinical evidence in T2-low asthma remains limited, current mechanistic and translational data provide a strong biological rationale for the further investigation of microbiota-targeted therapies in this challenging asthma endotype.

Future clinical practice is likely to move toward microbiome-guided precision nutrition and therapy, in which individual microbial and metabolic profiles inform personalized prevention and treatment strategies. The continued integration of microbiome science, nutritional research, and immunology will be essential for translating gut–lung axis insights into effective interventions for allergic asthma. In conclusion, elucidating gut microbiota-mediated immune and metabolic pathways highlights the gut–lung axis as a clinically relevant target, supporting the development of microbiome- and nutrition-informed strategies for asthma prevention and treatment.

## Figures and Tables

**Table 1:** Early-life factors shaping gut microbiota and asthma risk.

Early-Life Factor	Effect on Gut Microbiota	Immune Consequence	Association with Asthma Risk	References
Farm exposure	Increased microbial diversity; enrichment of environmental microbes	Enhanced innate immune training; increased immune tolerance	Strongly protective	[9,16,18]
Pet ownership in infancy	Increased household microbial richness	Promotes immune tolerance in high-diversity settings	Reduced risk with early exposure	[19,20]
Vaginal delivery	Early colonization by maternal vaginal and intestinal microbes	Supports normal immune maturation	Protective	[24]
Cesarean section	Delayed *Bacteroidetes* colonization; skin-associated microbes dominate	Impaired immune education	Modest association with increased asthma risk (attenuated in sibling analyses)	[25]
Breastfeeding	Promotes *Bifidobacterium* via HMOs	Induction of immune tolerance	Protective	[26]
Early-life antibiotics	Reduced microbial diversity; altered metabolism	Long-term immune dysregulation	Increased asthma susceptibility	[27]

HMOs: human milk oligosaccharides.

**Table 2:** Characteristics of gut microbiota dysbiosis in allergic asthma.

Dysbiosis Feature	Microbial Taxa or Trait	Direction	Clinical Relevance	References
α-diversity	Overall microbial richness	dec.	Ecosystem instability	[29]
SCFA producers	*F. prausnitzii*	dec.	Reduced anti- inflammatory capacity	[30,31]
Mucin degraders	*A. muciniphila*	dec.	Impaired gut barrier	[30]
Early-life commensals	*Bifidobacterium* spp.	dec.	Impaired immune tolerance	[39]
Proteobacteria expansion	*Escherichia coli*	inc.	Pro-inflammatory signature	[36]
Histamine producers	*Morganella* *morganii*	inc.	Elevated systemic histamine	[35]
Pro-allergic lipid production	Epoxide hydrolase genes	inc.	Increased 12,13-diHOME levels	[38]

SCFA: short-chain fatty acid; inc.: increase; dec.: decrease; 12,13-diHOME: 12,13-dihydroxy-9Z-octadecenoic acid.

**Table 3:** Microbial-derived metabolites mediating immune communication through the gut–lung axis.

Metabolite	Microbial Source	Host Target	Immunological Effect	Impact	References
Acetate	Fiber- fermenting bacteria	GPR43	Promotes Treg differentiation	Protective	[40]
Propionate	Fiber- fermenting bacteria	GPR41	Tolerogenic DCs; reduced ILC2	Protective	[40]
Butyrate	*F. prausnitzii*, *Clostridia*	HDAC inhibition	Suppresses Th2 cytokines	Protective	[43]
Indole derivatives (IAA, IPA)	*Lactobacillus* spp.	AhR	Enhances IL-22 production; maintains epithelial integrity	Protective	[47]
12,13- diHOME	Epoxide hydrolase–producing bacteria	Immune metabolic pathways	Reduces Treg number and function	Pro-allergic	[38]
Bacterial- derived histamine	*Morganella morganii*	H1/H4 histamine receptors	Promotes bronchoconstriction and inflammation	Pro-allergic	[35]

IAA: indole-3-acetic acid; IPA: indole-3-propionic acid; AhR: aryl hydrocarbon receptor; HDAC: histone deacetylase; Treg: regulatory T cell; 12,13-diHOME: 12,13-dihydroxy-9Z-octadecenoic acid.

**Table 4:** Experimental and translational strategies targeting the gut–lung axis.

Intervention	Primary Target	Evidence Type	Key Outcomes	Limitations	References
High-fiber diet	Endogenous SCFA production	Animal	Reduced airway inflammation; enhanced immune tolerance	Limited human data	[40]
Fermented foods	Gut microbiota modulation	Observational/limited clinical evidence	Potential immune modulation	Limited direct asthma evidence	[57]
Conventional probiotics	Gut microbiota composition	Human	No consistent evidence of clinical benefit	Strain-specific effects; poor colonization	[59,60]
Next- generation probiotics	Functional commensals (e.g., *F. prausnitzii*)	Preclinical	Restoration of anti-inflammatory metabolites	Manufacturing and stability challenges	[30,62]
Postbiotics	Microbial metabolites (SCFAs)	Preclinical	Direct immunomodulatory effects	Dosing and delivery unresolved	[40]
Fecal microbiota transplantation	Global microbiota restoration	Animal	Improved lung function; reduced inflammation and remodeling	Safety and standardization concerns	[46]

## Data Availability

The original contributions presented in this study are included in the article. Further inquiries can be directed to the corresponding authors.

## Abbreviations

The following abbreviations are used in this manuscript:

SCFA	short-chain fatty acid
Th2	T helper 2
HMOs	human milk oligosaccharides
URECA	Urban Environment and Childhood Asthma
ISAAC	International Study of Asthma and Allergies in Childhood
FLVR	*Faecalibacterium*, *Lachnospira*, *Veillonella*, and *Rothia*
Treg	regulatory T
HDAC	histone deacetylase
HDACs	histone deacetylases
IL	interleukin
AhR	aryl hydrocarbon receptor
12,13-diHOME	12,13-dihydroxy-9Z-octadecenoic acid
GPRs	G protein-coupled receptors

## References

[1] Kwah J.H., Peters A.T. (2019). Asthma in adults: Principles of treatment. Allergy Asthma Proc..

[2] Akar-Ghibril N., Casale T., Custovic A., Phipatanakul W. (2020). Allergic Endotypes and Phenotypes of Asthma. J. Allergy Clin. Immunol. Pract..

[3] Lambrecht B.N., Hammad H. (2015). The immunology of asthma. Nat. Immunol..

[4] Safiri S., Carson-Chahhoud K., Karamzad N., Sullman M.J.M., Nejadghaderi S.A., Taghizadieh A., Bell A.W., Kolahi A.A., Ansarin K., Mansournia M.A. (2022). Prevalence, Deaths, and Disability-Adjusted Life-Years Due to Asthma and Its Attributable Risk Factors in 204 Countries and Territories, 1990–2019. Chest.

[5] Cao Y., Chen S., Chen X., Zou W., Liu Z., Wu Y., Hu S. (2022). Global trends in the incidence and mortality of asthma from 1990 to 2019: An age-period-cohort analysis using the global burden of disease study 2019. Front. Public Health.

[6] Zhang Z.-Q., Li J.-Y., Fu C.-Y., Li Y.-L., Guo Q., Bao Y.-W., Wu J., Liao J.-C., Song Y.-Q., Li D.-X. (2025). Global, regional and national burden of asthma attributable to metabolic diseases from 1990 to 2021 and projected trends to 2040. BMC Pulm. Med..

[7] Holgate S.T. (2012). Innate and adaptive immune responses in asthma. Nat. Med..

[8] Pavord I.D., Beasley R., Agusti A., Anderson G.P., Bel E., Brusselle G., Cullinan P., Custovic A., Ducharme F.M., Fahy J.V. (2018). After asthma: Redefining airways diseases. Lancet.

[9] von Mutius E., Vercelli D. (2010). Farm living: Effects on childhood asthma and allergy. Nat. Rev. Immunol..

[10] Strachan D.P. (1989). Hay fever, hygiene, and household size. BMJ.

[11] Rook G.A. (2010). 99th Dahlem conference on infection, inflammation and chronic inflammatory disorders: Darwinian medicine and the ‘hygiene’ or ‘old friends’ hypothesis. Clin. Exp. Immunol..

[12] Haahtela T. (2019). A biodiversity hypothesis. Allergy.

[13] Ng Q.X., Yaow C.Y.L., Moo J.R., Koo S.W.K., Loo E.X.L., Siah K.T.H. (2024). A systematic review of the association between environmental risk factors and the development of irritable bowel syndrome. J. Gastroenterol. Hepatol..

[14] Riedler J., Braun-Fahrlander C., Eder W., Schreuer M., Waser M., Maisch S., Carr D., Schierl R., Nowak D., von Mutius E. (2001). Exposure to farming in early life and development of asthma and allergy: A cross-sectional survey. Lancet.

[15] Braun-Fahrlander C., Riedler J., Herz U., Eder W., Waser M., Grize L., Maisch S., Carr D., Gerlach F., Bufe A. (2002). Environmental exposure to endotoxin and its relation to asthma in school-age children. N. Engl. J. Med..

[16] Ege M.J., Mayer M., Normand A.C., Genuneit J., Cookson W.O., Braun-Fahrlander C., Heederik D., Piarroux R., von Mutius E., Group G.T.S. (2011). Exposure to environmental microorganisms and childhood asthma. N. Engl. J. Med..

[17] Loss G., Apprich S., Waser M., Kneifel W., Genuneit J., Buchele G., Weber J., Sozanska B., Danielewicz H., Horak E. (2011). The protective effect of farm milk consumption on childhood asthma and atopy: The GABRIELA study. J. Allergy Clin. Immunol..

[18] Stein M.M., Hrusch C.L., Gozdz J., Igartua C., Pivniouk V., Murray S.E., Ledford J.G., Marques Dos Santos M., Anderson R.L., Metwali N. (2016). Innate Immunity and Asthma Risk in Amish and Hutterite Farm Children. N. Engl. J. Med..

[19] Fall T., Lundholm C., Ortqvist A.K., Fall K., Fang F., Hedhammar A., Kampe O., Ingelsson E., Almqvist C. (2015). Early Exposure to Dogs and Farm Animals and the Risk of Childhood Asthma. JAMA Pediatr..

[20] Lynch S.V., Wood R.A., Boushey H., Bacharier L.B., Bloomberg G.R., Kattan M., O’Connor G.T., Sandel M.T., Calatroni A., Matsui E. (2014). Effects of early-life exposure to allergens and bacteria on recurrent wheeze and atopy in urban children. J. Allergy Clin. Immunol..

[21] Ver Heul A., Planer J., Kau A.L. (2019). The Human Microbiota and Asthma. Clin. Rev. Allergy Immunol..

[22] Gensollen T., Iyer S.S., Kasper D.L., Blumberg R.S. (2016). How colonization by microbiota in early life shapes the immune system. Science.

[23] Arrieta M.C., Stiemsma L.T., Dimitriu P.A., Thorson L., Russell S., Yurist-Doutsch S., Kuzeljevic B., Gold M.J., Britton H.M., Lefebvre D.L. (2015). Early infancy microbial and metabolic alterations affect risk of childhood asthma. Sci. Transl. Med..

[24] Dominguez-Bello M.G., Costello E.K., Contreras M., Magris M., Hidalgo G., Fierer N., Knight R. (2010). Delivery mode shapes the acquisition and structure of the initial microbiota across multiple body habitats in newborns. Proc. Natl. Acad. Sci. USA.

[25] Sevelsted A., Stokholm J., Bonnelykke K., Bisgaard H. (2015). Cesarean section and chronic immune disorders. Pediatrics.

[26] Milani C., Duranti S., Bottacini F., Casey E., Turroni F., Mahony J., Belzer C., Delgado Palacio S., Arboleya Montes S., Mancabelli L. (2017). The First Microbial Colonizers of the Human Gut: Composition, Activities, and Health Implications of the Infant Gut Microbiota. Microbiol. Mol. Biol. Rev..

[27] Russell S.L., Gold M.J., Hartmann M., Willing B.P., Thorson L., Wlodarska M., Gill N., Blanchet M.R., Mohn W.W., McNagny K.M. (2012). Early life antibiotic-driven changes in microbiota enhance susceptibility to allergic asthma. EMBO Rep..

[28] Petersen C., Round J.L. (2014). Defining dysbiosis and its influence on host immunity and disease. Cell. Microbiol..

[29] Abrahamsson T.R., Jakobsson H.E., Andersson A.F., Bjorksten B., Engstrand L., Jenmalm M.C. (2014). Low gut microbiota diversity in early infancy precedes asthma at school age. Clin. Exp. Allergy.

[30] Demirci M., Tokman H.B., Uysal H.K., Demiryas S., Karakullukcu A., Saribas S., Cokugras H., Kocazeybek B.S. (2019). Reduced Akkermansia muciniphila and Faecalibacterium prausnitzii levels in the gut microbiota of children with allergic asthma. Allergol. Immunopathol..

[31] Hu W., Lu W., Li L., Zhang H., Lee Y.K., Chen W., Zhao J. (2021). Both living and dead Faecalibacterium prausnitzii alleviate house dust mite-induced allergic asthma through the modulation of gut microbiota and short-chain fatty acid production. J. Sci. Food Agric..

[32] Fujimura K.E., Sitarik A.R., Havstad S., Lin D.L., Levan S., Fadrosh D., Panzer A.R., LaMere B., Rackaityte E., Lukacs N.W. (2016). Neonatal gut microbiota associates with childhood multisensitized atopy and T cell differentiation. Nat. Med..

[33] Stokholm J., Blaser M.J., Thorsen J., Rasmussen M.A., Waage J., Vinding R.K., Schoos A.M., Kunoe A., Fink N.R., Chawes B.L. (2018). Maturation of the gut microbiome and risk of asthma in childhood. Nat. Commun..

[34] Zheng P., Zhang B., Zhang K., Lv X., Wang Q., Bai X. (2020). The Impact of Air Pollution on Intestinal Microbiome of Asthmatic Children: A Panel Study. Biomed. Res. Int..

[35] Barcik W., Pugin B., Westermann P., Perez N.R., Ferstl R., Wawrzyniak M., Smolinska S., Jutel M., Hessel E.M., Michalovich D. (2016). Histamine-secreting microbes are increased in the gut of adult asthma patients. J. Allergy Clin. Immunol..

[36] Hilty M., Burke C., Pedro H., Cardenas P., Bush A., Bossley C., Davies J., Ervine A., Poulter L., Pachter L. (2010). Disordered microbial communities in asthmatic airways. PLoS ONE.

[37] Huang Y.J., Nelson C.E., Brodie E.L., Desantis T.Z., Baek M.S., Liu J., Woyke T., Allgaier M., Bristow J., Wiener-Kronish J.P. (2011). Airway microbiota and bronchial hyperresponsiveness in patients with suboptimally controlled asthma. J. Allergy Clin. Immunol..

[38] Levan S.R., Stamnes K.A., Lin D.L., Panzer A.R., Fukui E., McCauley K., Fujimura K.E., McKean M., Ownby D.R., Zoratti E.M. (2019). Elevated faecal 12,13-diHOME concentration in neonates at high risk for asthma is produced by gut bacteria and impedes immune tolerance. Nat. Microbiol..

[39] Stiemsma L.T., Arrieta M.C., Dimitriu P.A., Cheng J., Thorson L., Lefebvre D.L., Azad M.B., Subbarao P., Mandhane P., Becker A. (2016). Shifts in Lachnospira and Clostridium sp. in the 3-month stool microbiome are associated with preschool age asthma. Clin. Sci..

[40] Trompette A., Gollwitzer E.S., Yadava K., Sichelstiel A.K., Sprenger N., Ngom-Bru C., Blanchard C., Junt T., Nicod L.P., Harris N.L. (2014). Gut microbiota metabolism of dietary fiber influences allergic airway disease and hematopoiesis. Nat. Med..

[41] Budden K.F., Gellatly S.L., Wood D.L., Cooper M.A., Morrison M., Hugenholtz P., Hansbro P.M. (2017). Emerging pathogenic links between microbiota and the gut-lung axis. Nat. Rev. Microbiol..

[42] Dang A.T., Marsland B.J. (2019). Microbes, metabolites, and the gut-lung axis. Mucosal Immunol..

[43] Furusawa Y., Obata Y., Fukuda S., Endo T.A., Nakato G., Takahashi D., Nakanishi Y., Uetake C., Kato K., Kato T. (2013). Commensal microbe-derived butyrate induces the differentiation of colonic regulatory T cells. Nature.

[44] Arpaia N., Campbell C., Fan X., Dikiy S., van der Veeken J., deRoos P., Liu H., Cross J.R., Pfeffer K., Coffer P.J. (2013). Metabolites produced by commensal bacteria promote peripheral regulatory T-cell generation. Nature.

[45] Chang P.V., Hao L., Offermanns S., Medzhitov R. (2014). The microbial metabolite butyrate regulates intestinal macrophage function via histone deacetylase inhibition. Proc. Natl. Acad. Sci. USA.

[46] Lai Y., Qiu R., Zhou J., Ren L., Qu Y., Zhang G. (2025). Fecal Microbiota Transplantation Alleviates Airway Inflammation in Asthmatic Rats by Increasing the Level of Short-Chain Fatty Acids in the Intestine. Inflammation.

[47] Zelante T., Iannitti R.G., Cunha C., De Luca A., Giovannini G., Pieraccini G., Zecchi R., D’Angelo C., Massi-Benedetti C., Fallarino F. (2013). Tryptophan catabolites from microbiota engage aryl hydrocarbon receptor and balance mucosal reactivity via interleukin-22. Immunity.

[48] Dickson R.P., Erb-Downward J.R., Martinez F.J., Huffnagle G.B. (2016). The Microbiome and the Respiratory Tract. Annu. Rev. Physiol..

[49] Bisgaard H., Hermansen M.N., Buchvald F., Loland L., Halkjaer L.B., Bonnelykke K., Brasholt M., Heltberg A., Vissing N.H., Thorsen S.V. (2007). Childhood asthma after bacterial colonization of the airway in neonates. N. Engl. J. Med..

[50] Lai Y., Zhou J., Ren L., Qu Y., Xie X., Lai Y., Cao J., Li N., Qiu R., Liu M. (2026). Gut-lung axis: Moxibustion’s impact on short-chain fatty acids in various tissues of asthmatic rats. Int. Immunopharmacol..

[51] Goleva E., Jackson L.P., Harris J.K., Robertson C.E., Sutherland E.R., Hall C.F., Good J.T., Gelfand E.W., Martin R.J., Leung D.Y. (2013). The effects of airway microbiome on corticosteroid responsiveness in asthma. Am. J. Respir. Crit. Care Med..

[52] Green B.J., Wiriyachaiporn S., Grainge C., Rogers G.B., Kehagia V., Lau L., Carroll M.P., Bruce K.D., Howarth P.H. (2014). Potentially pathogenic airway bacteria and neutrophilic inflammation in treatment resistant severe asthma. PLoS ONE.

[53] Taylor S.L., Leong L.E.X., Choo J.M., Wesselingh S., Yang I.A., Upham J.W., Reynolds P.N., Hodge S., James A.L., Jenkins C. (2018). Inflammatory phenotypes in patients with severe asthma are associated with distinct airway microbiology. J. Allergy Clin. Immunol..

[54] Wypych T.P., Wickramasinghe L.C., Marsland B.J. (2019). The influence of the microbiome on respiratory health. Nat. Immunol..

[55] Suez J., Zmora N., Zilberman-Schapira G., Mor U., Dori-Bachash M., Bashiardes S., Zur M., Regev-Lehavi D., Ben-Zeev Brik R., Federici S. (2018). Post-Antibiotic Gut Mucosal Microbiome Reconstitution Is Impaired by Probiotics and Improved by Autologous FMT. Cell.

[56] Liu Y., Dai J., Zhou G., Chen R., Bai C., Shi F. (2025). Innovative Therapeutic Strategies for Asthma: The Role of Gut Microbiome in Airway Immunity. J. Asthma Allergy.

[57] Marco M.L., Heeney D., Binda S., Cifelli C.J., Cotter P.D., Foligne B., Ganzle M., Kort R., Pasin G., Pihlanto A. (2017). Health benefits of fermented foods: Microbiota and beyond. Curr. Opin. Biotechnol..

[58] Wastyk H.C., Fragiadakis G.K., Perelman D., Dahan D., Merrill B.D., Yu F.B., Topf M., Gonzalez C.G., Van Treuren W., Han S. (2021). Gut-microbiota-targeted diets modulate human immune status. Cell.

[59] Elazab N., Mendy A., Gasana J., Vieira E.R., Quizon A., Forno E. (2013). Probiotic administration in early life, atopy, and asthma: A meta-analysis of clinical trials. Pediatrics.

[60] Cuello-Garcia C.A., Brozek J.L., Fiocchi A., Pawankar R., Yepes-Nunez J.J., Terracciano L., Gandhi S., Agarwal A., Zhang Y., Schunemann H.J. (2015). Probiotics for the prevention of allergy: A systematic review and meta-analysis of randomized controlled trials. J. Allergy Clin. Immunol..

[61] Tang A.S.P., Quek J., Sulaimi F., Sim B., Kai T.O.S., Soon E.Y., Goh C.N., Hsu J.L.J., Lee C.K.L., Chong S.K.S. (2026). Probiotics for Irritable Bowel Syndrome: An Updated Systematic Review and Meta-Analysis With Trial Sequential Analysis. J. Gastroenterol. Hepatol..

[62] Debarry J., Garn H., Hanuszkiewicz A., Dickgreber N., Blumer N., von Mutius E., Bufe A., Gatermann S., Renz H., Holst O. (2007). Acinetobacter lwoffii and Lactococcus lactis strains isolated from farm cowsheds possess strong allergy-protective properties. J. Allergy Clin. Immunol..

[63] Kim Y.J., Bunyavanich S. (2025). Microbial influencers: The airway microbiome’s role in asthma. J. Clin. Investig..

[64] Yang Z., Mao W., Wang J., Yin L. (2025). The gut-lung axis in asthma: Microbiota-driven mechanisms and therapeutic perspectives. Front. Microbiol..

